# Demography: Fast and Slow

**DOI:** 10.1111/padr.12464

**Published:** 2022-01-14

**Authors:** Francesco C. Billari

**Keywords:** population turnover rate, migration share of turnover, fast demography, slow demography

## Abstract

Scientific ideas on the human population tend to be rooted in a “slow demography” paradigm, which emphasizes an inertial, predictable, self‐contained view of population dynamics, mostly dependent on fertility and mortality. Yet, demography can also move fast. At the country level, it is crucial to empirically assess how fast demography moves by taking migratory movements into account, in addition to fertility and mortality. We discuss these ideas and present new estimates of the speed of population change, that is, country‐level population turnover rates, as well as the share of turnover due to migration, for all countries in the world with available data between 1990 and 2020. Population turnover is inversely related to population size and development, and migratory movements tend to become important factors in shaping demography for both small and highly developed countries. Longitudinally, we analyze annual turnover data for Italy and Germany, documenting the changing speed of population change over time and its determinants. Accepting the “fast and slow” demography perspective has several implications for science and policy, which we discuss.

How do we make sense of human population change? Ideally, we start from a reliable snapshot of the present, based on solid data on the recent past and adequate knowledge about the more distant past. This information is used to understand the direction of population change and to feed future scenarios. Population data are organized in temporal scales that are considered to be appropriate according to the current scientific consensus: what temporal scale is appropriate might vary, also, within a subject. In meteorology, for instance, data are timed over decades and years or centuries, as well as over hours and minutes depending on the specific purpose of research and/or analysis. For instance, in weather forecasting, aggregating precipitation data at the hourly level implies higher information loss, as compared to the aggregation of air temperature data (Krzyszczak et al. 2017). As we will see, the same is true in demography: in certain times and places, population change is slow, and data can be gathered “slowly”; in other times and places, it becomes fast, and data also need to be gathered “fast.” Of course, there is a hierarchy, as fast data can be averaged to generate data over longer time scales, but the reverse is not possible. Realizing that population can change in a fast way, therefore, implies a change of paradigm in demographic measurement and data collection.

In what follows, we refer explicitly to the need for a paradigm shift that includes fast population change, as demographers have traditionally seen the “long run” for population change in contemporary times as referring to several decades, with the next few years inertially dependent on the present. Paul Demeny explicitly stated: “the near‐term future … in demographic matters, may be defined as the next 5 to 20 years” (Demeny 1984, 103). As a consequence, population scholars and experts have felt the confidence to issue long‐term population scenarios that span a number of decades. These widely used scenarios have been developed and regularly updated by supranational bodies, such as the United Nations Population Division with a biannual series of population prospects, Eurostat for the European Union, and a number of national‐level official agencies (Booth 2006). The related idea that population change materializes slowly, but with important and widespread consequences, has been clearly articulated by Alfred Sauvy (the founding director of the INED, the French Institute for Demographic Studies). In a series of essays, Sauvy has used the metaphor of a watch to describe the speed of demography, compared to other crucial components of societal change. In contrast with politics and the economy, which move with the “fast” hands of seconds and minutes, demography moves slowly, with the hour hand. Yet, “the short hand of the watch is the most important, even if it seems immobile” (Sauvy 1957, 5). In fact, many demographic indicators of reproduction measure change through a generational replacement lens, comparing a generation to the previous one, over a span of about 30 years. With regard to data collection, the 10‐year typical time window between population censuses, as well as the custom of estimating population quantities over five‐year intervals, for instance, is in line with this perspective.

Sauvy saw what we can define as demography's “slowness” as an opportunity to inform policymakers about potential responses to observed or foreseen population change. In this “slow demography” perspective, population moves inertially, and it is exogenous to other factors. In more recent terms, demography becomes a “megatrend” that drives global and local trends in a number of domains: economic, political, and social change; education; and climate change. Population scenarios can therefore be built as self‐contained: beyond general ideas on the direction of change according to the demographic transition, no background factors are taken into account when forecasting population trends in standard practice.

In this paper, we first review and assess from a general point of view this “slow demography” paradigm. We then introduce the need to explicitly take “fast demography” into account, using an empirical perspective and advocating the widespread use of two simple indicators based on the idea of population turnover. Indicators on population turnover have already been developed, but they have been so far underused to measure the speed of population change. We then estimate the recent and current levels of country‐level population turnover, and the increasing relevance of migratory movements, using the share of turnover due to migratory movements as an indicator of this relevance. Fast population turnover, with the rising contribution of migration, is particularly relevant for countries with smaller populations, but it has become increasingly more relevant for societies reaching the highest levels of the human development index (HDI), including countries with relatively large populations. Furthermore, we document the changing speed of population change over time for two cases: Italy and Germany. We conclude that demography is both “fast and slow.” We discuss the implications of this emerging perspective, in terms of scientific and data challenges for population, as well as its interrelations with other dimensions of global and local change, including policy issues.

## The “slow demography” paradigm

In current scientific discourse, the inertial and slow nature of demography is epitomized by two paradigmatic and interrelated global‐level megatrends: the demographic transition and population aging. The demographic transition from high mortality and fertility to low mortality and fertility is the main lens through which we can generally make sense of population change (Vallin 2002). While during the demographic transition population change is rapid (Bongaarts 2009), at the end of this process, population ideally reaches an equilibrium, with growth rates that are similar to the pretransitional situation, but without the “waste” of human lives implied by high mortality, and with a much slower pace of population change (Livi‐Bacci 2017).

The demographic transition is linked with the “slow” demography paradigm and its focus on long‐term predictability of population trends in at least three ways. First, it contrasts a pretransitional Malthusian “fast” world with high demographic turnover (punctuated by sudden epidemics, famines, wars, and baby booms and busts), with a posttransitional “slow” world with low demographic turnover (Livi‐Bacci 2017). Indeed, Thomas Malthus, in his 1798 *Essay*, already juxtaposed the problems of fast population growth with the virtues of slow population growth (Malthus 1798). Second, the demographic transition implies long‐term convergence for all populations at similar levels of low mortality and fertility (Wilson 2001). While global demographic convergence is still widely questioned, convergence within groups of societies is largely accepted, especially with regard to fertility and mortality, and this idea is used to inform long‐term scenarios (Castiglioni, Dalla‐Zuanna, and Tanturri 2020). Convergence is central to one of the key methodological approaches in demography, with an explicit focus on long‐term equilibrium, that is, stable population theory (Coale 1968). Third, the demographic transition is seen as a self‐sustained process that does not intrinsically depend on the evolution of nondemographic factors (Dyson 2010). For this reason, it provides a framework that can help generate long‐term demographic forecasts. Incorporating the demographic transition idea into statistical models, for instance, has allowed the generation of probabilistic population forecasts by the United Nations (Raftery et al. 2012).

Population aging is a direct consequence of the demographic transition, at both the global and the country level (Chesnais 1990). As humans become able to control their lives by improving their health and choosing freely and responsibly the number of children they have, middle‐ and later older‐age adults first emerge as a sizable component in populations. For a while, demographic windows of opportunity for economic growth open in societies where a large number of young and middle‐aged adults prevail, creating the potential for a demographic dividend (Bloom, Canning, and Sevilla 2003). Within the demographic transition scheme, the opening and closing of demographic windows of opportunities are seen as “slow” and predictable over decades. Population aging emerges in the last stage of the transition, with the rise of older adults, and is destined to become a global phenomenon.

Aging has also fostered new ideas on population change, such as the concept of “replacement migration,” popularized in a report by the United Nations (United Nations 2001). This concept focuses on the role of migration as a potential counterforce to population aging resulting from low fertility and low mortality. While there has been a general debate, with skeptical views on replacement migration (Coleman 2002), some empirical findings have documented that in low‐fertility countries such as Italy, migration has indeed slowed population aging over five‐ or 10‐year intervals (Billari and Dalla‐Zuanna 2011). Also using “slow demography” approaches based on stable populations, migration can be shown to slow population aging (Alho 2008). More generally, in terms of methods, population scholars have emphasized measures that can be interpreted over the long run, for instance, considering the total fertility rate and life expectancy as shaping intrinsic rates of growth and age distributions in stable populations. The gross and net reproduction ratios, which compare the size of a generation with the size of a previous one using fertility and mortality indicators, have been used to measure generational replacement, with the possibility of including migratory movements in this generational replacement idea (Billari and Dalla‐Zuanna 2013; Wilson et al. 2013).

The “slow demography” idea, that the demographic future does not generally depend on nondemographic factors, has also allowed the use of demographic scenarios and forecasts as inputs in scenarios for other factors. Considered the easiest factor to forecast, demography has therefore become a “forecasting tool” (Lindh 2003). Demographic scenarios have confidently been used to inform a variety of predictions, for instance, outlining scenarios on the future of the economy (Bloom et al. 2007); political and social change, democratization (Cincotta 2017), political identity (Lutz, Kritzinger, and Skirbekk 2006), religiosity and culture (Hackett et al. 2015); and education and climate change (O'Neill et al. 2010). With this opportunity also comes a risk: when demographers get it wrong, their errors propagate to scenarios that use demography as a forecasting tool.

## The speed of population change: Data and methods

While the “slow demography” paradigm does not predict fast population change in posttransitional societies, assessing the actual pace of change is an empirical matter. Yet, this matter is rarely dealt with, even if there is a readily available, and simple, measure of the speed of demography: the *population turnover rate* (PTR). Population turnover rates relate the total amount of flows (in and out) to the population of reference. While traditionally used in ecology (Schoener and Spiller 1987), organizational demography (Stewman 1988), and geography (Dieleman, Clark, and Deurloo 2000), these measures are surprisingly scarcely used to describe demographic dynamics, including in official statistics and standard sets of indicators on population change.

First of all, let us introduce the simple, formal definition of a *population turnover rate* (*PTR*), starting from its constituent components. For a given country j, within a specific time frame (0,t), and given estimates for the number of births $B_{j}\left(0,t\right)$, the number of deaths $D_{j}\left(0,t\right)$, and average population size (or person‐years lived) $P_{j}\left(0,t\right)$, the (crude annual) birth rate and the (crude annual) death rate are respectively:

$$b_{j}\left(0,t\right)=\frac{B_{j}\left(0,t\right)}{t\cdot P_{j}\left(0,t\right)}$$

$$d_{j}\left(0,t\right)=\frac{B_{j}\left(0,t\right)}{t\cdot P_{j}\left(0,t\right)}$$

Given the number of immigrants and emigrants, $I_{j}\left(0,t\right)$ and $E_{j}\left(0,t\right)$, the (crude annual) immigration rate and the (crude annual) emigration rate are, respectively:

$$i_{j}\left(0,t\right)=\frac{I_{j}\left(0,t\right)}{t\cdot P_{j}\left(0,t\right)}$$

$$e_{j}\left(0,t\right)=\frac{E_{j}\left(0,t\right)}{t\cdot P_{j}\left(0,t\right)}.$$

The PTR is then defined as:

$$PTR_{j}\left(0,t\right)=b_{j}\left(0,t\right)+d_{j}\left(0,t\right)+i_{j}\left(0,t\right)+e_{j}\left(0,t\right)$$

A fundamental indicator to characterize the nature of population change is the *migration share of turnover* (*MST*), which can be computed using either rates or absolute numbers, and is—by definition—between 0 and 100 percent:

$$MST_{j}\left(0,t\right)=\frac{i_{j}\left(0,t\right)+e_{j}\left(0,t\right)}{PTR_{j}\left(0,t\right)}=\frac{I_{j}\left(0,t\right)+E\left(0,t\right)}{B_{j}\left(0,t\right)+D_{j}\left(0,t\right)+I_{j}\left(0,t\right)+E_{j}\left(0,t\right)}.$$

For a given population, in a specific period of time, the PTR summarizes the speed of population change using the basic flow rates of population dynamics. A major challenge in estimating country‐level PTRs is related to data quality and availability: while birth and death rates are widely available over time and space, reliably estimating data on immigration and emigration flows at the country level is difficult (Abel and Cohen 2019; Abel and Sander 2014). This difficulty might also explain the lack of adequate attention, so far, to the speed of population change. The most relevant work on the impact of migration on population change has been focused on internal migration, where population turnover has been explicitly used as a measure by Dennett and Stillwell (2008). Martin Bell and coauthors (Bell et al. 2015) use net migration measures to compare internal migration intensities across countries. Philip Rees and coauthors (Rees et al. 2017) develop measures of the impact of internal migration at the national level that relate net migration to a measure of turnover, assessing the “efficiency” of migration in shaping change in populations. Andrei Rogers (1990) documented the widespread focus of scholars and agencies on net migration, an idea that obscures the behavioral differences between immigration and emigration, and that does not, therefore, allow one to grasp the role of migration in shaping population change.

At the global level, thanks to new bilateral migration flow estimates provided by Abel and Cohen (2019), combined with UN birth and death rate estimates, it is possible to estimate national‐level turnover rates. Therefore, in our subsequent global analyses, data on birth rates, death rates, and average population for the five‐year periods 1990–1995, 1995–2000, 2000–2005, 2005–2010, 2010–2015, and 2015–2020 are obtained from the UN World Population Prospects (UNWPP) database (United Nations, Department of Economic and Social Affairs, Population Division 2019). To estimate PTR, migration flows are needed for each country. UNWPP only provides estimates about net migrations. We, therefore, use estimated bilateral international migration flows reconstructed for 200 countries by Guy Abel and Joel Cohen; aggregating bilateral flows (Abel and Cohen 2019); and in particular the updated estimates provided in version 5 by Guy Abel, based on UN International Migrant Stock Data 2020 and UNWPP 2019.^1^ Total estimated flows to a country provide the number of immigrants $I_{j}\left(0,t\right)$, while total flows from a country provide the number of emigrants $E_{j}\left(0,t\right)$. For our main results, we select Abel–Cohen estimates based on the “demographic account pseudo Bayesian closed” method, which provides the largest correlation coefficient with “ground truth” validation sets for outmigration rates (0.44), and the second‐largest correlation coefficient for immigration rates (0.82). As a robustness check (see online Appendix: Supplemental Materials), we also produce estimates based on the “demographic account minimization closed” approach by Abel–Cohen. The latter approach provides the highest correlation with immigration rates in their validation exercise (0.84), but a substantially lower correlation with outmigration rates (0.30).

At the national level, some statistical offices produce estimates of population flows that include immigration and emigration, also on an annual basis. For our case studies, we use official estimates provided by the national statistical institutes of Germany (DESTATIS—*Federal Statistical Office*, data between 1990 and 2020) and Italy (ISTAT—*Italian National Institute of Statistics*, data between 1916 and 2020) (note that for both Germany and Italy, the 2020 data we use are still considered provisional).

## Global‐level results: Population turnover rates and the share due to migratory movements

How fast is demography, actually? We first deal with the population of the world. In this case, by definition, migratory rates are zero, and the world PTR is simply determined by the sum of birth and death rates, that is, PTR = *b* + *d*. Using UN estimates, the turnover rate of the world population has more than halved over the course of two‐thirds of a century: from 56 per 1000 for the 1950–1955 time period to 26 per 1000 for the 2015–2020 time interval (United Nations, Department of Economic and Social Affairs, Population Division 2019). This decrease of the world‐level speed of population change, with a rather regular decline over almost seven decades, is in line with the prediction linked to the demographic transition of a movement from fast to slow demography. Using estimates for pretransitional birth and death rates (Reher 2004), one can infer that PTRs without migration were, in “normal times” as high as about 80 per 1000 in societies that are “latecomers” in the demographic transition process (mostly in Africa and Asia). Peaks in pretransitional turnover rates are likely to have been reached during years of mortality crises in the pre‐Industrial world. Goldstein (2015) formally proved that, in a population closed to migratory flows, PTR is lowest when population growth is negative. Therefore, while global population aging is going to possibly increase the death rate, the continuation of the decline in the birth rate may imply a further, but limited, reduction of the world's population turnover. Indeed, the United Nations “medium” variant scenario (base 2019) projects *t* at 23 per 1000 for 2095–2100.

**FIGURE 1:**
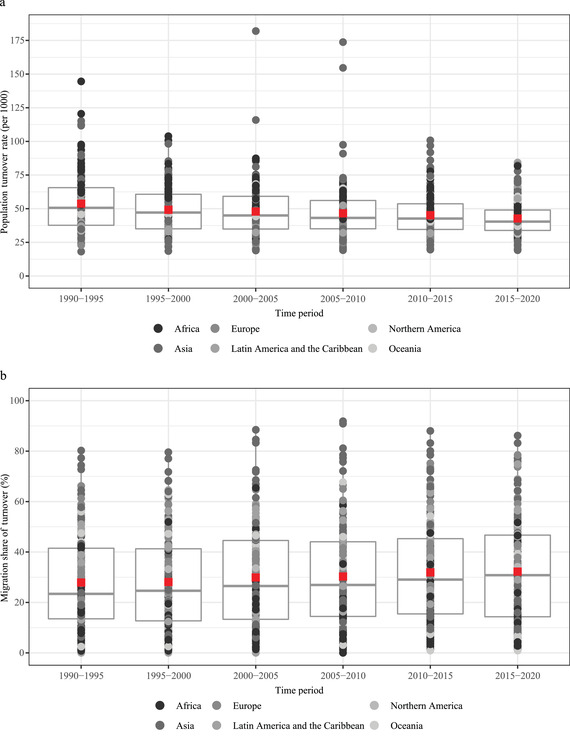
Country‐level annual PTR (per 1000) (a) and MST (%) (b). Five‐year periods between 1990 and 2020. In the box plots, one point is a country; the box shows the first quartile, median, and third quartile, and the red square point is the (unweighted) average SOURCE: Own elaborations on UN WPP2019 and Abel and Cohen (2019 and updates).

We now turn to country‐level results, therefore including migration flows. Figure 1(a) shows the box plots for the distribution of annual estimates of country‐level PTRs for five‐year intervals between 1990–1995 and 2015–2020. It documents the important heterogeneity in the speed of population change across countries, within a general downward trend for the average speed. Indeed, the average country‐level PTR is 52.7 per 1000 for 1990–1995 and 42.8 for 2015–2020 (data not weighted by population).^2^ These trends are not homogeneous across regions, however. For instance, within the same period, the average country‐level PTR has increased from 37.9 to 39.5 per 1000 on average in Europe and decreased from 67.1 to 47.6 in Africa. The relevance of migratory movements in turnover rates at the country level can be assessed through the MST. A significant threshold, here, is at 50 percent: above this threshold, migratory movements constitute the prevalent source of demographic change. Box plots of MST are shown in Figure 1(b), documenting both the heterogeneity across countries and an overall increase over time. The average country‐level percentage share of turnover due to migration is 27.6 percent for 1990–1995 and 32.2 percent for 2015–2020 (data not weighted by population).^3^ Again, trends are heterogeneous over regions: MST has increased over this period from 35.8 to 45.0 percent on average in Europe, and it has decreased from 18.6 to 16.8 percent in Africa.

**FIGURE 2:**
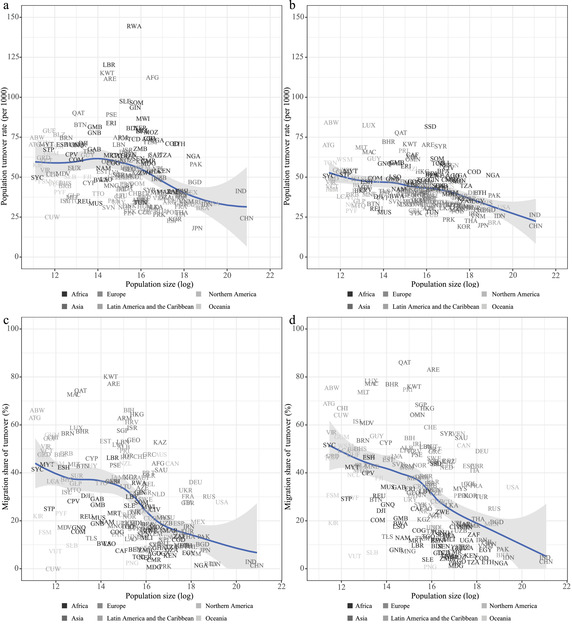
Country‐level annual PTR (per 1000) for 1990–1995 (a) and 2015–2020 (b), and MST (%) for 1990–1995 (c) and 2015–2020 (d), by country size. Loess smoothing and 95 percent confidence bands. For country labels, see the Supporting Information SOURCE: Own elaborations on UN WPP2019 and Abel and Cohen (2019 and updates).

The speed of demography is related to country‐level population scale: in Figure 2, we document the cross‐sectional relationship between PTR, MST, and population size (using a logarithmic scale) for 1990–1995 and 2015–2020. The negative association between PTR and population size, already noticeable for 1990–1995, becomes evident for 2015–2020. This relationship is steeper when we consider MST: migratory movements tend to be at least as important as births and deaths in smaller countries. For 2015–2020, for countries with a population size of less than about 15 million, the average MST is around 50 percent.

**FIGURE 3:**
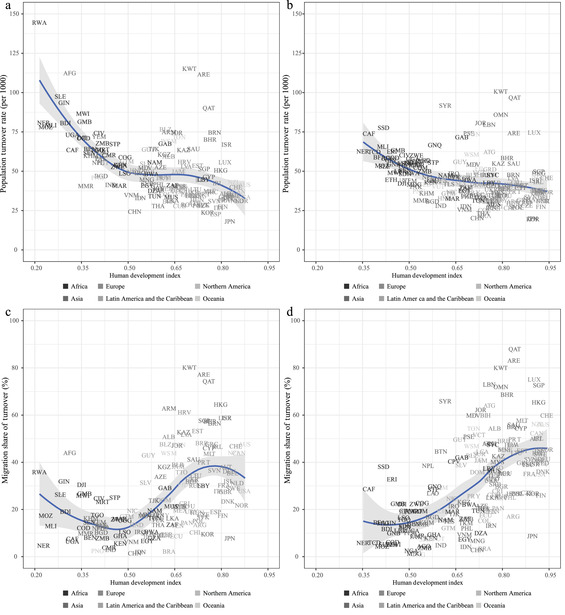
Country‐level annual PTR (per 1000) for 1990–1995 (a) and 2010–2015 (b), and MST (%) for 1990–1995 (c) and 2010–2015 (d), by HDI. Loess smoothing and 95 percent confidence bands. For country labels, see the Supporting Information SOURCE: Own elaborations on UN WPP2019, Abel and Cohen (2019 and updates), and UNDP.

The idea of demographic transition is intrinsically linked to development. Indeed, analyses of demographic transitions have compared population change to societal development, as measured by income, or more comprehensive measures such as the HDI devised by the United Nations (Bongaarts and Watkins 1996; Myrskylä, Kohler, and Billari 2009). In Figure 3, we show the country‐level relationships between PTR, MST, and the HDI (for which data are available until 2010–2015). A negative relationship, which is visible for 1990–1995, is less noticeable for 2010–2015. For the latter data point, turnover rates reach a plateau at higher levels of the HDI. This pattern is linked to the increasing role of migratory movements in shaping PTR. Figure 3(d), in particular, documents a clear positive relationship between MST and HDI.

## Fast and slow demography over time: The cases of Italy and Germany

Our global, country‐level analyses have shown that while in posttransitional societies, demography tends to be slower as society develops, faster demography seems to reemerge and is definitely not rare among countries with advanced levels of development. To explain this (re)emergence of “fast” demography after the demographic transition, we need to give up the paradigmatic idea that population change tends to be self‐contained, inertial, and therefore exogenous to other factors. We must thus focus on factors that can influence population turnover in the short run: when the rate of change is fast, demography can also move fast.

Fast population change might happen for several factors. First, the economy. Since Malthus first noted this connection, economic developments, including sudden recessions and crises, or booms, have affected key components of population change. Recent evidence confirms this link, for example, for fertility (Sobotka, Skirbekk, and Philipov 2011) and migration (Massey 1988). However, given that economic crises can have opposite effects on fertility and migration, for instance, it is hard to make a general prediction on their association with the speed of population change. Technology, often proceeding through discoveries in a discontinuous fashion, can also significantly affect population dynamics, as shown by Ester Boserup (Boserup 1976). Technological and medical discoveries have also shaped fertility through the contraceptive revolution that led to a “second demographic transition” (Lesthaeghe 2014). More recently, the fast‐paced digital revolution has shaped several health and fertility outcomes (Rotondi et al. 2020) as well as migration (Pesando et al. 2021) and might trigger faster population change.

Politics affects demography in a number of ways that are also potentially fast‐moving, that is, policies; sudden political discontinuities, such as the end of a political regime and the beginning of a new one; and wars (Livi‐Bacci 2021). Political shifts may imply fast shifts in all of the components of population change: fertility, migration, and mortality. Likewise, wars are extreme cases of political decisions quickly affecting fertility (e.g., Blanc 2004; Cetorelli 2014), mortality (e.g., Li and Wen 2005), and migration (e.g., Abel et al. 2019). Demographic behaviors, and migration, in particular, are part of the adaptative responses to climate change impacts, and environmental crises and disasters often play a role in the acceleration of population change (Hunter, Luna, and Norton 2015). The Covid‐19 pandemic has compressed deaths in a short time span compared to other epidemics of the posttransitional world (Goldstein and Lee 2020) and in general has contributed to focusing our attention on fast demographic change (Zagheni 2021). The decline in international mobility during Covid‐19, together with potential increases in the number of deaths (and decreases in the number of births), has an ambiguous effect on turnover, while for a pandemic‐affected year, one can expect a decline in the share of turnover due to migration.

We now explore the role of different factors shaping population turnover by focusing on two case studies. At the global level, we analyzed the speed of population change from a cross‐sectional perspective. Longitudinally, it would be possible to have insights from single‐country analyses of the data we built using the five‐year interval estimates between 1990–1995 and 2015–2020 available for each country. Nevertheless, the need to fully assess the changing speed of demography over time ideally requires more fine‐grained data, at least on an annual basis. Thanks to data provided by the national statistical offices, which include explicit information on immigration and emigration, we show here two case studies using yearly data: Italy and Germany.

**FIGURE 4:**
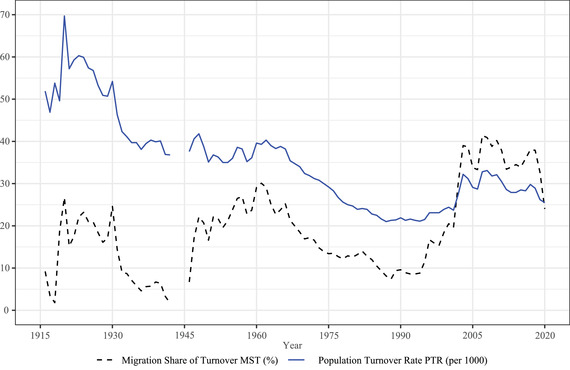
Annual PTR (per 1000) (blue, solid line), MST (%) (black, dashed line), Italy, 1916–2020 (with the exception of 1943–1945 during World War II) SOURCE: Own elaborations on Italian National Institute of Statistics (ISTAT) data.

For Italy, it is possible to estimate PTR and MST for slightly over a century, between 1916 and 2020 (Figure 4, data are missing during the last years of World War II, 1943–1945). Until 1995, emigration and immigration data only referred to Italian nationals, so that both PTR and MST are underestimated; still, given the low levels of foreign migration to Italy until the mid‐1990s (0.6 percent of the resident population in Italy had foreign citizenship according to the 1991 census), this underestimate is likely not dramatic. During the last part of the nineteenth century and the first years of the twentieth century, before the period analyzed here, the demographic transition, with its substantial mortality and fertility declines, is linked with considerable emigration to Northern Europe and the Americas in particular. Our data start in the middle of World War I. The Great War and the Spanish flu are major *ancien régime* crises for Italian demography, with the combination of the two crises leading to record death rates and absolute numbers in 1918 (Glei, Bruzzone, and Caselli 2005). The post–World War I emigration boom is linked to the highest level of PTR (69.7 per 1000) in our data, for 1920, with MST at 26.7 percent. Afterward, population turnover starts a slow decline, maintaining a substantial share of migration. Emigration control becomes a policy target under the Fascist regime, which had a particular interest in population policies, including formal restrictions on departures in 1926 (Cannistraro and Rosoli 1979; Ipsen 1996). The *ventennio*, about two decades of Fascist rule, linked with the decision to enter World War II, and its high mortality leads to a PTR of 36.8 per 1000 and an MST of 1.7 percent in 1942.

After World War II, during the economic boom connected to reconstruction, Italy's PTRs remain stable around 35–40 per 1000, with a substantial increase to levels between 20 percent and 30 percent as a result of a new wave of outmigration (MST peaks at 30.1 percent in 1960) for about two decades. The subsequent decline in emigration, linked with the significant fertility decline that eventually turned Italy into a “lowest‐low fertility” forerunner (Kohler, Billari, and Ortega 2002), leads to minimum levels of PTR, which reaches about 21–22 per 1000 between 1987 and 1995 (with MST lower than 10 percent). Indeed, as we noted, 1995 is a source of discontinuity in our data, since until then immigration and emigration data refer only to Italian citizens.

After 1995, the speed of population change starts to increase again. The rise of both PTR and MST is mostly linked with a massive immigration boom, with a period of immigration booms and busts, and Italy clearly becoming a destination country for international migrants (Colombo and Dalla‐Zuanna 2019). PTR climbs back until the year of the Great Recession, particularly dramatic for Italy, reaching 33.1 per 1000 in 2008. The peak in MST is reached in 2007 (41.4 percent). The long economic crisis after the Great Recession has an important impact on Italy's demography. The booms and busts in population turnover after 2008 are visible in the roller‐coaster dynamics of MST, while PTR remains fluctuating but relatively stable until the outbreak of Covid‐19. Indeed, 2020, the year Covid‐19 spread to Italy, sees a decrease in PTR to 25.5 per 1000, despite a substantial rise in the number of deaths (+17.6 percent compared to 2019), while births decline by 3.8 percent. This decrease is, for the most part, the result of the fall of emigration, and even more so of immigration, with MST in 2020 at 24 percent, its lowest level since 2001.

Over this century, the speed of population change in Italy has been affected first by either the tail of the demographic transition and its links to mass emigration or by epidemics such as the Spanish flu and Covid‐19. Yet, political decisions have fundamentally shaped the pace of population change: the decision to participate in world wars is the most impactful one (particularly for its terrible effects on mortality) on population turnover. In contrast, family policies and migration policies have been rather passive, one could say *laissez faire*, at least after World War II (Colombo and Dalla‐Zuanna 2019).

**FIGURE 5:**
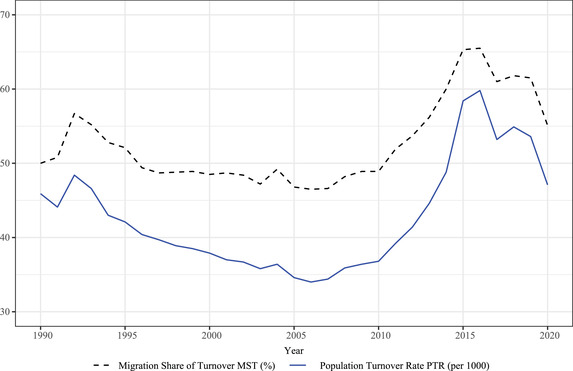
Annual PTR (per 1000) (blue, solid line) and MST (%) (black, dashed line), Germany, 1990–2020 SOURCE: Own elaborations on German Federal Statistical Office (DESTATIS) data.

The case of another posttransitional, wealthy, and relatively large‐population country, epitomizes the impact of sudden political and policy changes on demography. Germany, with more than 83 million inhabitants in 2020, is the most populated country among the top 10 in terms of the HDI (where, at 0.947, it ranks sixth overall). During the period subsequent to German reunification (1990 onward), East Germany was significantly impacted by the fall of fertility and outmigration (Kreyenfeld and Vatterrott 2018). After the election of Chancellor Angela Merkel, in 2005, policies on migrants and refugees were changed in important ways. For instance, during the 2015 refugee crisis, kickstarted by the civil war in Syria, an initially prudent Chancellor Merkel refused “to set an upper limit on the number of refugees Germany would accept” (Helms, Esch, and Crawford 2019, 359). The speed of population change after reunification therefore first declines, and then accelerates significantly. Figure 5 documents these trends, by showing estimates of Germany's PTR and MST between 1990 and 2020. Up to 2006, fertility decline and a low contribution of migration bring annual turnover to its lowest level of 34.0 per 1000 in 2006. The subsequent increase is because of a small recovery in fertility, and above all, to the contribution of migratory movements. PTR peaks after Chancellor Merkel's 2015 decision, reaching 59.8 per 1000 in 2016, with 65.5 percent due to migration. In 2020, with the Covid‐19 pandemic also spreading to Germany, immigration rates record a substantial decline. Despite the increase in deaths, and in the same direction as the case of Italy, the deceleration of immigration leads to a lower turnover (47.1 per 1000), and a decline in the share due to migration (55.1 percent). Our analyses show that “fast” German demography during these three decades has therefore been shaped substantially by political decisions as well as by the decrease in migration during the Covid‐19 pandemic.

## Conclusions and implications

Demography is both slow and fast. Using the PTR, we have shown that the global demographic transition, with its associated and widespread declines in mortality and fertility, has brought about a significant slowing down of population turnover at the world level, as predicted by theory. At the country level, on the contrary, demography can be fast and become even faster even in populations that are wealthy and have completed the demographic transition. Moreover, using the MST, we have shown that migratory movements can be key determinants of fast population change at the country level. The changing speed of population change in posttransitional societies, with the crucial role of migration, has become evident when inspecting estimates of annual country‐level PTRs and MSTs for our two cases: Italy and Germany. MST is linked to both (lower) population size and levels of development when analyzing countries at the global level and to political and economic factors for Italy and Germany. The role of migration in shaping the speed of population change has so far not received adequate attention, perhaps for the (endogenous) lack of systematic data on immigration and emigration flows, but also for the longer‐term perspective usually adopted in the “replacement migration” debate.

In general, we can conclude that the currently prevalent “slow demography” perspective is misleading when applied as a general approach to the study of population change. This is especially true when one focuses on the country level (or below). Taking a “fast demography” perspective that complements the “slow” one is therefore crucial for the years ahead. This has important implications for science and policy—some of which are of a more speculative nature—which we now address.

With regard to population science, the implications of a “fast and slow demography” perspective are multiple. First, demographic data collection needs to fully take the speed of population change into account, collecting more up‐to‐date and frequent information on stocks and flows at higher frequencies, as opposed to relying on more traditional snapshots such as population censuses, or surveys, spaced 10 years apart. We have new opportunities thanks to the digital revolution: more frequent information on population and enhanced methods for “nowcasting” the present using register data as well as digital footprints (Cesare et al. 2018; Fiorio et al. 2021). For instance, the use of daily mortality surveillance data has already been central to studying air pollution (Liu et al. 2019) and Covid‐19 (Michelozzi et al. 2020), and Facebook data at fine spatio‐temporal granularity have been used to show the impact of Hurricane Maria on emigration from Puerto Rico (Alexander, Polimis, and Zagheni 2019). Research on the use of digital information to document migration, fertility, and mortality can help boost knowledge in a fast‐changing population environment, with the opportunity to also provide open, real‐time access.

Second, demographic theories need to become more aware of the scale of the population they are discussing and to focus more on the determinants and consequences of the speed of population change. Elegant theoretical constructs and implied empirical analyses in demography are too often based on explicit or implicit assumptions of zero migration. Moreover, the traditional focus on net migration (i.e., the difference between the number of immigrants and the number of outmigrants) has hidden the relevance of migratory flows for demography (Rogers 1990). While the relevance of migratory flows in shaping population change has been documented at the level of cities (Lerch 2020), we have shown that it is also relevant for many societies and that this relevance might change over time.

Third, the practice of making long‐term population forecasts, which relies on the “slow demography” inertial idea, might mislead users, especially at the national level. While the acknowledgment of uncertainty can be made explicit in probabilistic population forecasts (Raftery et al. 2012), levels of uncertainty are unlikely to be adequately estimated given the huge uncertainty at the country level, especially concerning migratory movements (Azose, Ševčíková, and Raftery 2016).

Fourth, a fast demography perspective underscores the fact that population change is not exogenous to other factors, but that it coevolves with them. Using demography as a “forecasting tool” comes at a potentially too high cost, especially at the country level, as other factors may also influence demography in the short run. The evolution of these factors, therefore, needs to be jointly considered. A promising proposal to deal with the need for drawing scenarios is to explicitly consider the coevolution of factors, including demography, in plausible pathways. This has been done in the context of climate change research, where “shared socioeconomic pathways” have been devised as “reference pathways describing plausible alternative trends in the evolution of society and ecosystems over a century timescale, in the absence of climate change or climate policies” (O'Neill et al. 2014).

Fifth, scholars need to pay more attention to the determinants of demographic behavior, and the potential fast shifts in these determinants. Behavioral theories that complement the typical macro‐oriented view of demography with a microlevel foundation are crucial in this effort, and the speed of change within individual life courses also deserves scientific attention.

A “fast and slow demography” perspective also has important policy implications. First, in terms of science and data collection policy, more funding needs to be directed toward a more frequent and fine‐grained collection of data on population issues, including migratory flows. Demographic statistics also need to partly bridge the gap with economic statistics, which are collected more frequently, for what concerns fertility and mortality. Data collection on population issues (including surveys) needs to become more integrated with the factors interacting with demographic behavior. Second, institutions, both at the supranational and the country level, need to be aware that demography can change fast. Policymakers and experts advising them should acknowledge the uncertainty surrounding long‐term demographic scenarios, and the impact that they could have on population change, including in the short run. As an example of political uses of demographic scenarios, in the runup to the Brexit referendum (November 2015), the British Prime Minister David Cameron wrote a letter to the President of the European Commission Donald Tusk. The letter used long‐term population forecasts as a political argument (“we are forecast to become the most populous country in the EU by 2050”), but also referred to failures in shorter‐term forecasting regarding (“… the current very high level of population flows from within the European Union into the United Kingdom. These have been unplanned and are much higher than forecast”). Third, when designing population policies, as in the Italian and German cases, more emphasis needs to be devoted to migratory movements, also tackling population aging from a replacement migration perspective. Fast demography is less inertial than slow demography, and it matters.

## Supporting information


**Figure A1**. Country‐level annual Population Turnover Rates PTR (per 1000) (a), and Migration Share of Turnover MST (%) (b). 5‐year periods between 1990 and 2020
**Figure A2**. Country‐level annual Population Turnover Rates PTR (per 1000) for 1990‐95 (a) and 2015‐20 (b), and Migration Share of Turnover MST (%) for 1990‐95 (c) and 2015‐20 (d), by country size. Loess smoothing and 95% confidence bands
**Figure A3**. Country‐level annual Population Turnover Rates PTR (per 1000) for 1990‐95 (a) and 2010‐15 (b), and Migration Share of Turnover MST (%) for 1990‐95 (c) and 2010‐15 (d), by Human Development Index (HDI
Figure A4, A5, and A6 reproduce the results of Figure 3 in the main paper using, separately, the non‐demographic components of the UNDP Human Development Index, i.e. income (measured as Gross National Income per capita, adjusted for purchasing power), and education (measured both using expected years of schooling, and mean years of schooling)
**Figure A4**. Country‐level annual *PTR* (per 1000) for 1990‐95 (a) and 2010‐15 (b), and *MST* 1990‐95 (c) and 2010‐15 (d), by Gross National Income (log) per capita
**Figure A5**. Country‐level annual *PTR* (per 1000) for 1990‐95 (a) and 2010‐15 (b), and *MST* 1990‐95 (c) and 2010‐15 (d), by expected years of schooling
**Figure A6**. Country‐level annual *PTR* (per 1000) for 1990‐95 (a) and 2010‐15 (b), and *MST* 1990‐95 (c) and 2010‐15 (d), by mean years of schooling. Loess smoothing and 95% confidence bands

## Data Availability

Data on birth rates, death rates, immigration rates, emigration rates, and the derived estimates of PTR and MST used for the paper are available through *figshare* (https://doi.org/10.6084/m9.figshare.16751869).

## Notes

1 See https://guyabel.com/publication/bilateral‐international‐migration‐flow‐estimates/#version‐5‐update‐for‐ims2020 (downloaded on August 12, 2021).

2 When weighted by log‐population size (to reduce the weight of extreme cases), the average PTR declines from 53.7 per 1000 in 1990–1995 to 42.8 in 2015–2020.

3 When weighted by log‐population size (to reduce the weight of extreme cases), the average MST increases from 27.8 percent in 1990–1995 to 32.1 percent in 2015–2020.
