# Supplementary material for: Demography: Fast and Slow
Source: Popul Dev Rev. 2022 Jan 14;48(1):9–30. doi: 10.1111/padr.12464 (PMC9305154; doi:10.1111/padr.12464)
Supplement: Supplementary file 1 — Figure A1. Country‐level annual Population Turnover Rates PTR (per 1000) (a), and Migration Share of Turnover MST (%) (b). 5‐year periods between 1990 and 2020 Figure A2. Country‐level annual Population Turnover Rates PTR (per 1000) for 1990‐95 (a) and 2015‐20 (b), and Migration Share of Turnover MST (%) for 1990‐95 (c) and 2015‐20 (d), by country size. Loess smoothing and 95% confidence bands Figure A3. Country‐level annual Population Turnover Rates PTR (per 1000) for 1990‐95 (a) and 2010‐15 (b), and Migration Share of Turnover MST (%) for 1990‐95 (c) and 2010‐15 (d), by Human Development Index (HDI Figure A4, A5, and A6 reproduce the results of Figure 3 in the main paper using, separately, the non‐demographic components of the UNDP Human Development Index, i.e. income (measured as Gross National Income per capita, adjusted for purchasing power), and education (measured both using expected years of schooling, and mean years of schooling) Figure A4. Country‐level annual PTR (per 1000) for 1990‐95 (a) and 2010‐15 (b), and MST 1990‐95 (c) and 2010‐15 (d), by Gross National Income (log) per capita Figure A5. Country‐level annual PTR (per 1000) for 1990‐95 (a) and 2010‐15 (b), and MST 1990‐95 (c) and 2010‐15 (d), by expected years of schooling Figure A6. Country‐level annual PTR (per 1000) for 1990‐95 (a) and 2010‐15 (b), and MST 1990‐95 (c) and 2010‐15 (d), by mean years of schooling. Loess smoothing and 95% confidence bands [file PADR-48-9-s001.docx]

**Demography: Fast and Slow**

**Francesco C. Billari***

**Appendix: Supplemental Materials**

This file includes supplemental materials for the article ‘Demography: Fast and Slow’, published in *Population and Development Review*. In particular, Appendix A includes a number of figures, which reproduces the ones of the main manuscript using a different method for the reconstruction of migration flows as a robustness check. In Appendix B the results of Figure 3, on the relationship between the Population Turnover Rates (*PTR*) and the Migration Share of Turnover (*MST*) and the Human Development Index (*HDI*) are reproduced by using the non-demographic components of the *HDI*. Appendix C reports a brief description of the data used in the article, which are made available through *figshare*.

**Acknowledgments**

The author gratefully acknowledges Luca Badolato for extremely precious research assistance. This project has received important comments and suggestions, at various stages, from the DisCont group meeting at Bocconi, as well as from participants to the third Jan M. Hoem Distinguished Lecture, Stockholm University Demography Unity, to the 12^th^ Edition of the Dutch Demography Day, and to seminars at the Maryland Population Center and at the University of Wisconsin-Madison. This project has received funding from the European Research Council (ERC) under the European Union’s Horizon 2020 research and innovation program (grant agreement n° 694262), project DisCont—Discontinuities in Household and Family Formation (PI: F. C. Billari).

*Department of Social and Political Sciences and “Carlo F. Dondena” Centre for Research on Social Dynamics and Public Policy, via Röntgen 1, 20136 Milano, Italy. E-mail: francesco.billari@unibocconi.it

**Appendix A: Robustness checks on migration flows and components of the Human Development Index**

In this section, we reproduce the main analyses of the paper using alternative estimates for migration flows, and the (non-demographic) components of the Human Development Index separately. The general findings of our analyses remain stable with respect to these robustness checks.

First of all, we use Abel and Cohen’s “Demographic Account Pseudo Bayesian Closed” method for the estimation of migration flows (Abel and Cohen 2019),with the subsequent updates published by Guy Abel using International Migrant Stock (2020) and United Nations World Population Prospects 2019 (United Nations, Department of Economic and Social Affairs, Population Division 2019). For the updates, specifically, see <https://guyabel.com/publication/bilateral-international-migration-flow-estimates/#version-5-update-for-ims2020> (downloaded on 12 August 2021). Figures A1, A2, and A3 reproduce respectively Figure 1, 2, and 3 of the main paper.

**Figure A1.** Country-level annual Population Turnover Rates PTR (per 1000) (a), and Migration Share of Turnover MST (%) (b). 5-year periods between 1990 and 2020. In the boxplots, one point is a country, the box shows the first quartile, median, and third quartile, and the red square point is the (unweighted) average. Source: own elaborations on UN WPP2019 and Abel and Cohen (2020), using the “Demographic Account Pseudo Bayesian Closed” method for the estimation of migration flows.

**Figure A2**. Country-level annual Population Turnover Rates PTR (per 1000) for 1990-95 (a) and 2015-20 (b), and Migration Share of Turnover MST (%) for 1990-95 (c) and 2015-20 (d), by country size. Loess smoothing and 95% confidence bands. For country labels see Appendix C. Source: own elaborations on UN WPP2019 and Abel and Cohen (2020) , using the “Demographic Account Pseudo Bayesian Closed” method for the estimation of migration flows.

**Figure A3.** Country-level annual Population Turnover Rates PTR (per 1000) for 1990-95 (a) and 2010-15 (b), and Migration Share of Turnover MST (%) for 1990-95 (c) and 2010-15 (d), by Human Development Index (HDI). For country labels see Appendix C. Source: own elaborations on UN WPP2019, Abel and Cohen (2020), and UNDP, using the “Demographic Account Pseudo Bayesian Closed” method for the estimation of migration flows.

Figure A4, A5, and A6 reproduce the results of Figure 3 in the main paper using, separately, the non-demographic components of the UNDP Human Development Index, i.e. income (measured as Gross National Income per capita, adjusted for purchasing power), and education (measured both using expected years of schooling, and mean years of schooling). Figure A4 shows the cross-country relationship between *PTR*, *MST*, and the logarithm of Gross National Income (GNI) per capita, adjusted for purchasing power to 2017 US dollars. Figure A5 shows the cross-country relationship between *PTR*, *MST*, and expected years of schooling. Figure A6 shows the cross-country relationship between *PTR*, *MST*, and mean years of schooling.

**Figure A4.** Country-level annual *PTR* (per 1000) for 1990-95 (a) and 2010-15 (b), and *MST* 1990-95 (c) and 2010-15 (d), by Gross National Income (log) per capita. Loess smoothing and 95% confidence bands. For country labels see Appendix C. Source: own elaborations on UN WPP2019, Abel and Cohen (2019 and update), and UNDP.

**Figure A5.** Country-level annual *PTR* (per 1000) for 1990-95 (a) and 2010-15 (b), and *MST* 1990-95 (c) and 2010-15 (d), by expected years of schooling. Loess smoothing and 95% confidence bands. For country labels see Appendix C. Source: own elaborations on UN WPP2019, Abel and Cohen (2019 and update), and UNDP.

**Figure A6.** Country-level annual *PTR* (per 1000) for 1990-95 (a) and 2010-15 (b), and *MST* 1990-95 (c) and 2010-15 (d), by mean years of schooling. Loess smoothing and 95% confidence bands. For country labels see Appendix C. Source: own elaborations on UN WPP2019, Abel and Cohen (2019 and update), and UNDP.

**Appendix B: list of data files**

Here is a brief description of the files containing birth rates, death rates, immigration rates, emigration rates, and the derived estimates of *PTR* and *MST* used for the paper. The data (in .csv format) can be downloaded from *figshare* (doi:10.6084/m9.figshare.16751869).

.

- The file *Data_paper.csv* contains birth, death, immigration and emigration rates, and the derived estimates of country-level *PTR* and *MST* used in the paper (five-year intervals between 1990-95 and 2015-20), using Abel-Cohen estimates based on the “Demographic Account Pseudo Bayesian Closed” method.
- The file *Data_robust.csv* is equivalent to *Data_paper.csv* but it is based on Abel-Cohen “Demographic Account Minimisation Closed” estimates of migratory flows.
- The file *Data_labels.csv* contains the country labels used in Figures 2 and 3 in the main paper.
- The files *Data_Italy.csv* and *Data_Germany.csv* contain respectively the data on birth, death, immigration and emigration rates, and the estimates of annual *PTR* and *MST* for Italy and Germany.

**References**

Abel, Guy J., and Joel E. Cohen. 2019. “Bilateral International Migration Flow Estimates for 200 Countries.” *Scientific Data* 6 (1): 82. https://doi.org/10.1038/s41597-019-0089-3.

United Nations, Department of Economic and Social Affairs, Population Division. 2019. “World Population Prospects 2019, Online Edition. Rev. 1.” 2019. https://population.un.org/wpp/Download/Standard/Population/.
